# COVID-19 clinico-radiological mismatch: a proposal for a novel combined morphologic/volumetric CT severity score with blinded validation

**DOI:** 10.1186/s43055-021-00486-1

**Published:** 2021-04-15

**Authors:** Ahmed Samir, Abdelaziz Elnekeidy, Heba Said Gharraf, Ayman Ibrahim Baess, Tarek El-Diasty, Dina Altarawy

**Affiliations:** 1 Department of Radio-diagnosis, Faculty of Medicine, Alexandria University, Alexandria, Egypt; 2 Department of Chest Diseases, Faculty of Medicine, Alexandria University, Alexandria, Egypt; 3 Department of Radiology, Urology and Nephrology Center, Mansoura University, Mansoura, Egypt

**Keywords:** COVID-19, CTSI, Morphologic, Volumetric, Quantitative

## Abstract

**Background:**

Some COVID-19 patients with similar quantitative CT measurements had variable clinical presentation and outcome. The absence of reasonable clinical explanations, such as pre-existing comorbidities or vascular complications, adds to the confusion. The authors believed that neglecting the impact of certain severe morphologic features could be an alternative radiological explanation. This study aims to optimize the initial CT staging of COVID-19 and propose a new combined morphologic/volumetric CT severity index (CTSI) to solve this clinico-radiological mismatch.

**Results:**

This multi-center study included two major steps. The first step of the study entailed a standardized combined morphologic/volumetric CT severity analyses to propose a new optimized CTSI. This was conducted retrospectively during the period from June till September 2020. It included 379 acutely symptomatic COVID-19 patients. They were clinically classified according to their oxygen saturation and respiratory therapeutic requirements into three groups: group A (mild 298/79%), group B (borderline severity 57/15%), and group C (severe/critical 24/6%). The morphologic and volumetric assessment of their HRCT was analyzed according to severity, by two consultant radiologists in consensus. A new 25 point-CTSI has been created, combining eight morphological CT patterns [M1:M8; 8 points] and four grades of volumetric scores [S1:S4; 17 points]. The addition of the M5 pattern (air bubble sign), M6 pattern (early fibrosis and architectural distortion), or M7 pattern (crazy-paving) proved to increase the clinical severity. The second step of the study entailed a standardized blinded/independent validation analysis for the proposed CTSI. This was prospectively conducted on other 132 patients during October 2020 and independently performed by other two consultant radiologists. Validation results reached 80.2% sensitivity, 91.8% specificity, AUROC-curve = 0.8356, and 90.9% accuracy.

**Conclusion:**

A new optimized CTSI with accepted validation is proposed for initial staging of COVID-19 patients, using combined morphologic/volumetric assessment instead of the quantitative assessment alone. It could solve the clinico-radiological mismatch among patients with similar quantitative CT results and variable clinical presentation during the absence of pre-existing comorbidities or vascular complications.

## Background

On March 12, WHO announced COVID-19 as a pandemic [1, 2]^.^ Progression to ARDS and coagulation dysfunction was reported in severe cases [3]^.^ Chest computed tomography (CT) plays an important role in the initial evaluation and follow up of COVID-19 patients because of its high sensitivity [4–6]. The CT findings may also precede the onset of symptoms while the PCR screening may show false-negative results [7]. The European Society of Radiology and the European Society of Thoracic imaging recommended CT pulmonary angiography (CTPA) when non-enhanced CT failed to explain the severity of the respiratory failure [8]^.^

Several CT severity scores were introduced for COVID-19; all were based on manual or automated quantitative evaluation alone. Meanwhile, some COVID-19 patients with similar quantitative CT measurements had variable clinical presentation and outcome. The absence of any clinical explanations, such as pre-existing comorbidities or vascular complications, adds to this confusion.

The authors believed that neglecting the impact of certain severe morphologic features could be an alternative radiological explanation for this mismatch and suggested that the combination between volumetric and morphologic assessment is mandatory. Consequently, the authors performed this study to optimize COVID-19 initial CT staging and propose a new combined morphologic/volumetric CT severity index (CTSI) to solve the clinico-radiological mismatch among patients without comorbidities or vascular complications.

## Methods

### Study protocol and human population

This multi-center study was collectively conducted on 511 acutely symptomatic patients proved with COVID-19 during the period from June till October 2020. It was approved by the Institutional Ethics Committee. Patient consent was waived by the Research Ethics Board, assuring the respect of the confidentiality of patient’s data and medical records. The manuscript has no overlap with any previously published work.

Inclusion criteria were acutely symptomatic COVID-19 patients (during the 10 days from the onset of first clinical complaint) with positive PCR results and complete medical records.

Exclusion criteria were (1) asymptomatic patients, (2) incomplete medical records, (3) degraded quality of CT images with respiratory motion artifacts, (4) patients with known explanation for the clinical-radiological mismatch (48 patients were already excluded before the onset of the study) such as (A) pre-existing cardio-pulmonary or extra-pulmonary comorbidities such as emphysema, interstitial lung diseases, lung cancer, morbid obesity, co-existing neurological, cardiac, or abdominal diseases. All can impact the patient original O_2_ saturation and yield more deterioration of his condition not proportionate to the degree of lung involvement. (B) Vascular complications proved by CT pulmonary angiography among borderline-severe or severe/critical patients, such as acute pulmonary embolism.

### Clinical evaluation

The clinical evaluation was performed by two consultant pulmonologists (having long time experience in the field of chest diseases; 18–20 years).

Patients were clinically classified according to the clinical symptoms, oxygen saturation, and respiratory therapeutic requirements into three groups [9]:

- Group [A] (mild): patients with 95–100% O_2_ saturation/room-air (RA), absent or type I dyspnea, respiratory rate (RR) < 30/min, and no need for O_2_ support.
- Group [B] (borderline severe): patients with 93–94% O_2_ saturation/RA plus type II or III dyspnea and/or tachypnea (RR ≥ 30/min) or both.
- Group [C] (severe/critical): patients with < 93% O_2_ saturation/RA up to ARDS.
  The patients in group [B] and [C] could be indicated for initial high flow nasal oxygen therapy up to mechanical ventilation.

### CT machines and scanning parameters:

Multiple MDCT machines were used: (1) SOMATOM Sensation 64, Siemens Medical Systems, Germany, (2) Canon Medical Systems; Toshiba Aquilion 64, USA, and (3) Canon Medical Systems; Toshiba Aquilion CXL/CX 128, USA.

CT scanning parameters were slice thickness: 1–1.25 mm, tube rotation: 0.6–0.9 s, detector collimation 1 mm, 120–130 kVp, and 200 mA, FOV = 350 mm × 350 mm. Intravenous contrast administration was not used.

### Study design and steps

The study included two major steps:

1. *First step*: standardized combined morphological/volumetric CT severity analyses and proposal of a new CTSI.
2. *Second step*: standardized blinded/independent validation analysis for the proposed new CTSI.

#### Step (1): standardized combined morphological/volumetric CT severity analyses and proposal of a new CTSI

It was conducted retrospectively during the period from June till September 2020. It included 379 acutely symptomatic COVID-19 patients. They were 242 males and 137 females (63.9%:36.1%). Their age ranged from 10 to 80 years (mean age 45.42 ± 20.1 SD)*.* They were clinically classified into three groups: Group [A] (mild 298/79% patients). Group [B] (borderline severity 57/15% patients). Group [C] (severe or critical 24/6% patients).

CT images were analyzed in consensus with the availability of medical records by two expert consultant radiologists (having long time experience in thoracic imaging; 15 and 25 years). They performed combined CT volumetric and morphologic assessment followed by severity analysis and a new combined CTSI proposal.

[I] Volumetric/quantitative assessment [size of lesions (S)]

OsiriX MD 11.0 software (Pixmeo SARL, Geneva, Switzerland) was utilized for imaging review and volumetric/quantitative assessment of all patients, so the variability of the used MSCT machines would not impact the quantitative assessment. It was utilized for automated calculation of the total and pathological lung volumes based on threshold interval adjustment during the region of interest (ROI) 2D/3D color-coded reconstruction. (0:− 1024 Hu) the interval was set for total lung volume calculation and (0: − 700 Hu) interval was almost set for pathological lung volume calculation. Lesions were classified into four grades:

- [S1] Patchy lesions involving (< 15% of lung volume),
- [S2] Patchy lesions involving (15–25% of lung volume),
- [S3] Patchy lesions involving (25–50% of lung volume),
- [S4] Diffuse lung involvement (> 50% of lung volume).
  [II] Morphologic assessment (M)

Eight morphologic HRCT patterns were traced:

- [M1] Pure ground-glass opacities (GGOs) or solid nodules with peri-focal GG (halo sign).
- [M2] GGOs with a peripheral organization “Atoll or reversed halo sign.”
- [M3] GGOs mixed with consolidative changes.
- [M4] Homogeneous or “curvilinear” consolidations; the latter is parallel to the pleural lining.
- [M5] GGOs with “air bubble sign”; are small air-filled spaces within the ground-glass or consolidative changes, representing a cut section of sub-segmental bronchiolectasis sequel to fibrosis with focal air trapping.
- [M6] GGOs with “early secondary fibrotic changes” and architectural distortion; are irregular linear streaks and atelectatic plates, representing early scarring of cellular components, which could be associated with bronchial wall thickening and bronchiectatic changes.
- [M7] Patchy GGOs with smooth septal thickening “crazy-paving pattern,”
- [M8] Diffuse alveolar damage (DAD) pattern, showing diffuse lung involvement with either of the above-mentioned CT patterns or mixed (notably crazy-paving pattern and air bubble sign).

Extra-parenchymal HRCT findings were also assessed, including significant nodal enlargement (short axis > 1 cm) and pleuro-pericardial effusions.

The overall time for this combined quantitative and morphologic CT assessment was estimated and ranged from 6 to 12 min.

[III] Statistical severity analyses and CTSI proposal

- ❖ Prevalence of HRCT characteristics among the total number of patients.
- ❖ Statistical analysis of significant relation between each HRCT volumetric or morphologic characteristic and clinical severity. Chi-square tests and *P* value measurements were performed using an online calculator (https://www.socscistatistics.com). *P* value (< 0.05) was considered statistically significant.
- ❖ Calculation of the severity ratio for each HRCT abnormality among each group from the following equation [Severity ratio = Prevalence rate of HRCT finding among the total number of each patients’ group/Prevalence rate of each patients' group among total sample size]. The total severity index is then summed and the CTSI model is created.

#### Step (2): standardized blinded/independent validation analysis for the proposed new CTSI

It was conducted prospectively during October 2020 on other 132 patients. They included 72 males and 60 females (54.5%:45.5%). Their age ranged from 22 to 67 years (mean age 41.13 ± 11.69 SD). CT images were analyzed independently by two expert consultant radiologists (having long time experience in thoracic imaging; 10 and 30 years). They were blinded from the clinical records.

The following statistical methods were utilized:

- ❖ Inter-observer agreement (IOA) using Cohen’s Kappa test.
- ❖ Prevalence, sensitivity, specificity, positive predictive value (PPV), and negative predictive value (NPV), using an online diagnostic test calculator (http://araw.mede.uic.edu/cgi-bin/testcalc.pl).
- ❖ Receiver operating characteristics (ROC) curve, area under the curve (AUC), confidence interval, and accuracy, using QI Macros 2020 Excel software.

## Results

**Fig. 1:**
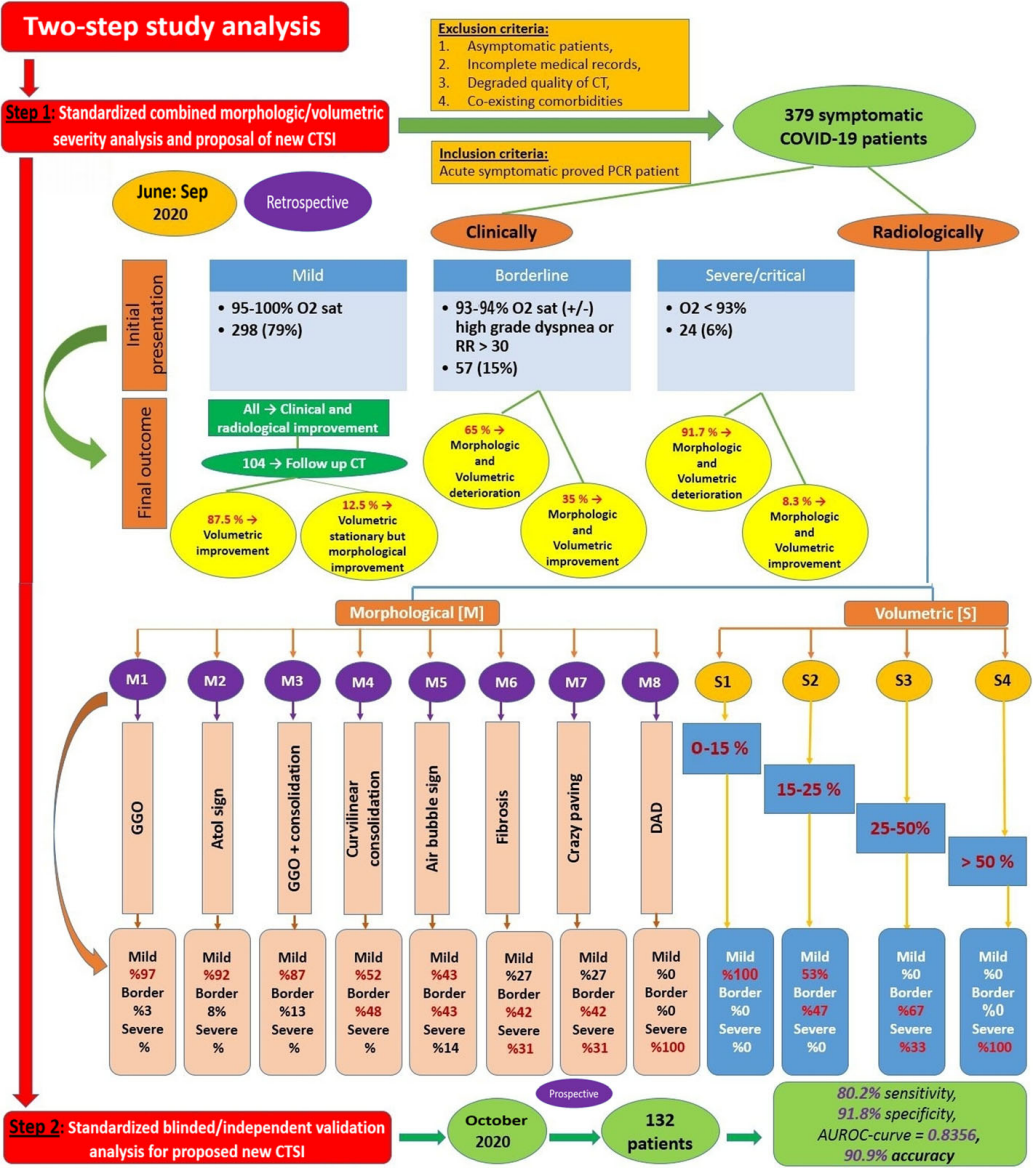
A flow diagram design summarizing the study steps and results

A flow diagram is demonstrating the study methodology including patients’ selection, study design, and steps together with final clinical and radiological results (Fig. 1).

### Step (1): standardized combined morphological/volumetric CT severity analyses and proposal of a new CTSI

#### Size of the lesions [S] with volumetric/quantitative assessment

**Fig. 2:**
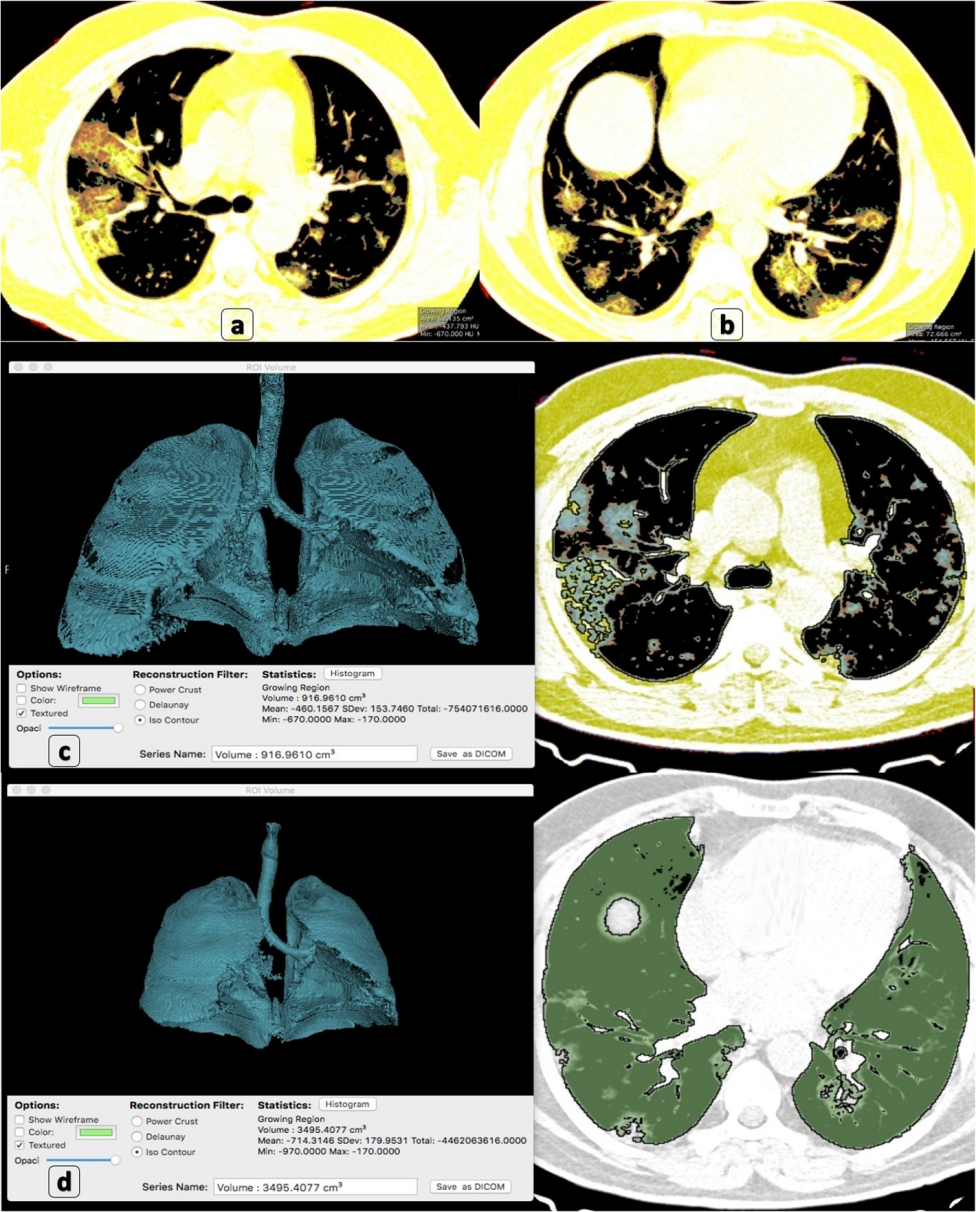
Volumetric/quantitative assessment using OsiriX MD 11.0 software: A 57-year-old male patient proved with COVID-19. **a**, **b** Osirix 2D computed volumetric analysis (axial cuts) show bilateral lower lobar sub-pleural patchy GGOs with “crazy-paving” reaching peri-hilar zones proximally. **c** Osirix 3D computed volumetric analysis after threshold interval adjustment shows 917 cm^3^ affected volume of the lung. **d** Osirix 3D computed volumetric analysis after threshold interval adjustment shows 3495 cm^3^ total lung volume

A significant relation between the size of the lesions and clinical severity was statistically proved (*P* values ranged from 0.00017 to < 0.0001) (Table 1). Prevalence and severity analysis was performed and detailed in (Table 2). [S1] lesions were the highest prevalent (72%) and the least severe (1.3). On the other hand, [S4] lesions were the least prevalent (2%) and the most severe (16.7). Examples of volumetric assessment are demonstrated (Fig. 2).

**Table 1:** Statistical analysis for significant relation between morphologic/volumetric HRCT findings and clinical severity

HRCT findings:		Mild (observed and expected values)	Borderline-severe and severe/critical (observed and expected values)	Chi-square after Yates correction	***P*** value
• **Volumetric analysis:**					
**[S1]**	**0–15% of lung volume is involved:**	271 (213.08)	0 (57.92)	258.4958	*< 0.00001*
**[S2]**	**15–25% of lung volume is involved:**	27 (40.10)	24 (10.90)	23.1384	*0.000017*
**[S3]**	**25–50% of lung volume is involved:**	0 (38.53)	49 (10.47)	216.8037	*< 0.00001*
**[S4]**	**> 50% of lung volume is involved:**	0 (6.29)	8 (1.71)	30.0667	*< 0.00001*
• **Morphologic pattern:**					
**[M1]**	**Pure GGO or solid nodule with GG halo sign:**	150 (121.09)	5 (32.91)	54.4123	*< 0.00001*
**[M2]**	**GGOs with “Atoll sign”:**	50 (42.46)	4 (11.54)	7.3078	*0.00687*
**[M3]**	**GGOs mixed with consolidations:**	70 (62.90)	10 (17.10)	4.7499	*0.02930*
**[M4]**	**Homogeneous consolidations or “curvilinear” consolidations:**	15 (22.80)	14 (6.20)	13.5262	*0.00024*
**[M5]**	**GGOs with “air bubble sign”:**	3 (5.50)	4 (1.50)	5.4304	*0.0198*
**[M6]**	**GGOs with early secondary fibrosis and architectural distortion:**	7 (20.44)	19 (5.56)	44.4097	*< 0.00001*
**[M7]**	**Patchy GGOs with smooth septal thickening “focal crazy paving”:**	7 (20.44)	19 (5.56)	44.4097	*< 0.00001*
**[M8]**	**It refers to diffuse alveolar damage pattern (DAD);** - **By definition, it is diffused (> 50%) of lung volume involvement [S4]** - **By definition, it represents mixed GGOs with “crazy-paving pattern” and “air bubble sign.”**				
		0 (5.5)	7 (1.5)	26.2377	*< 0.00001*
• **Other relevant signs:**					
*Others*	**Pleural effusion (minimal):**	8 (10.22)	5 (2.78)	2.3396	0.1261
*Others*	**Pericardial effusion (minimal):**	8 (7.08)	1 (1.92)	0.5776	0.4472
*Others*	**Tree in bud nodules:**	3 (3.15)	1 (0.85)	0.0317	0.8587

*All *P* value are < 0.05, considered as statistically significant

**Table 2:** Distribution of patients according to clinical severity and HRCT characteristics with estimation of proportionate severity factor

Clinical severity (respiratory status):				Group (1) mild			Group (2) border line severity			Group (3) severe–critical			Estimation of “total severity factor" and approximated score points	
- - -	O_**2**_ saturation and requirements: Dyspnea: Respiratory rate (RR):			95–100%, Absent or mild (type I) RR < 30/min			93–94% (+) one of: High grade (type II or III) RR ≥ 30/min			< 93% (O_**2**_ support or ventilation) High grade (type II or III) RR ≥ 30/min				
-	Total number (TN) and percentage (379)			298 (79%)			57 (15%)			24 (6%)				
HRCT characteristics:				***N***	%	^a^ ***Severity*** ***ratio***	***N***	%	***Severity*** ***ratio***	***N***	%	***Severity*** ***ratio***	Total	Points
• **Volumetric analysis:**														
**[S1]**	**0–15% of lung volume is involved:**	*Total:*	***271 (72%)***	271	100%	^a^ 1.3	Negative			Negative			**1.3**	**1**
**[S2]**	**15–25% of lung volume is involved:**	*Total:*	***51 (13%)***	27	53%	0.7	24	47%	3.1	Negative			**3.8**	**4**
**[S3]**	**25–50% of lung volume is involved:**	*Total:*	***49 (13%)***	Negative			33	67%	4.5	16	33%	5.5	**10**	**10**
**[S4]**	**> 50% of lung volume is involved:**	*Total:*	***8 (2%)***	Negative			Negative			8	100%	16.7	**16.7**	**17**
• **Morphologic pattern:**														
**[M1]**	**Pure GGO:**	*Total:*	***154 (41%)***	150	97%	1.2	4	3%	0.2	Negative			**1.4**	**1**
**[M2]**	**GGOs with “Atoll sign”:**	*Total:*	***50 (13%)***	46	92%	1.2	4	8%	0.5	Negative			**1.7**	**2**
**[M3]**	**GGOs mixed with consolidations:**	*Total:*	***80 (21%)***	70	87%	1.1	10	13%	0.9	Negative			**2**	**2**
**[M4]**	**Homogeneous consolidations or “curvilinear” consolidations**:	*Total:*	***29 (8%)***	15	52%	0.7	14	48%	3.2	Negative			**3.9**	**4**
**[M5]**	**GGOs with “air bubble sign”:**	*Total:*	***7 (2%)***	3	43%	0.5	3	43%	2.9	1	14%	2.3	**5.8**	**6**
**[M6]**	**GGOs with early secondary fibrosis and architectural distortion:**	*Total:*	***26 (7%)***	7	27%	0.3	11	42%	2.8	8	31%	5.2	**8.3**	**8**
**[M7]**	**Patchy GGOs with smooth septal thickening “focal crazy-paving” pattern:**	*Total:*	***26 (7%)***	7	27%	0.3	11	42%	2.8	8	31%	5.2	**8.3**	**8**
													**Maximum (SF = 25)**	
**[M8]**	**It refers to diffuse alveolar damage pattern (DAD);** - **By definition, it is diffused (> 50%) of lung volume involvement [S4]** - **By definition, it represents mixed GGOs with “crazy-paving pattern” and “air bubble sign.”**													
		*Total:*	***7 (2%)***	Negative			Negative			7 (100%)				

^a^ Severity ratio = prevalence rate of HRCT finding among the total number of each patients’ group/prevalence rate of each patients’ group among total sample size; example 100%/79% = 1.3

#### Morphological CT patterns [M]

A significant relation between morphological HRCT patterns and clinical severity was statistically proved (*P* values ranged from 0.02930 to < 0.0001) (Table 1). Prevalence and severity analysis was performed and detailed in (Table 2). [M1] lesions were the most prevalent (41%) and the least severe (1.4). On the other hand, [M5] lesions were the least prevalent (2%) and [M6] with [M7] lesions were the most severe (8.3 each).

Trivial percentages of other extra-parenchymal CT signs were depicted and detailed in (Table 1). No significant relation to clinical severity could be statistically proved (*P* values ranged from 0.1261 to 0.8587).

#### Combined volumetric and morphologic analysis

**Fig. 3:**
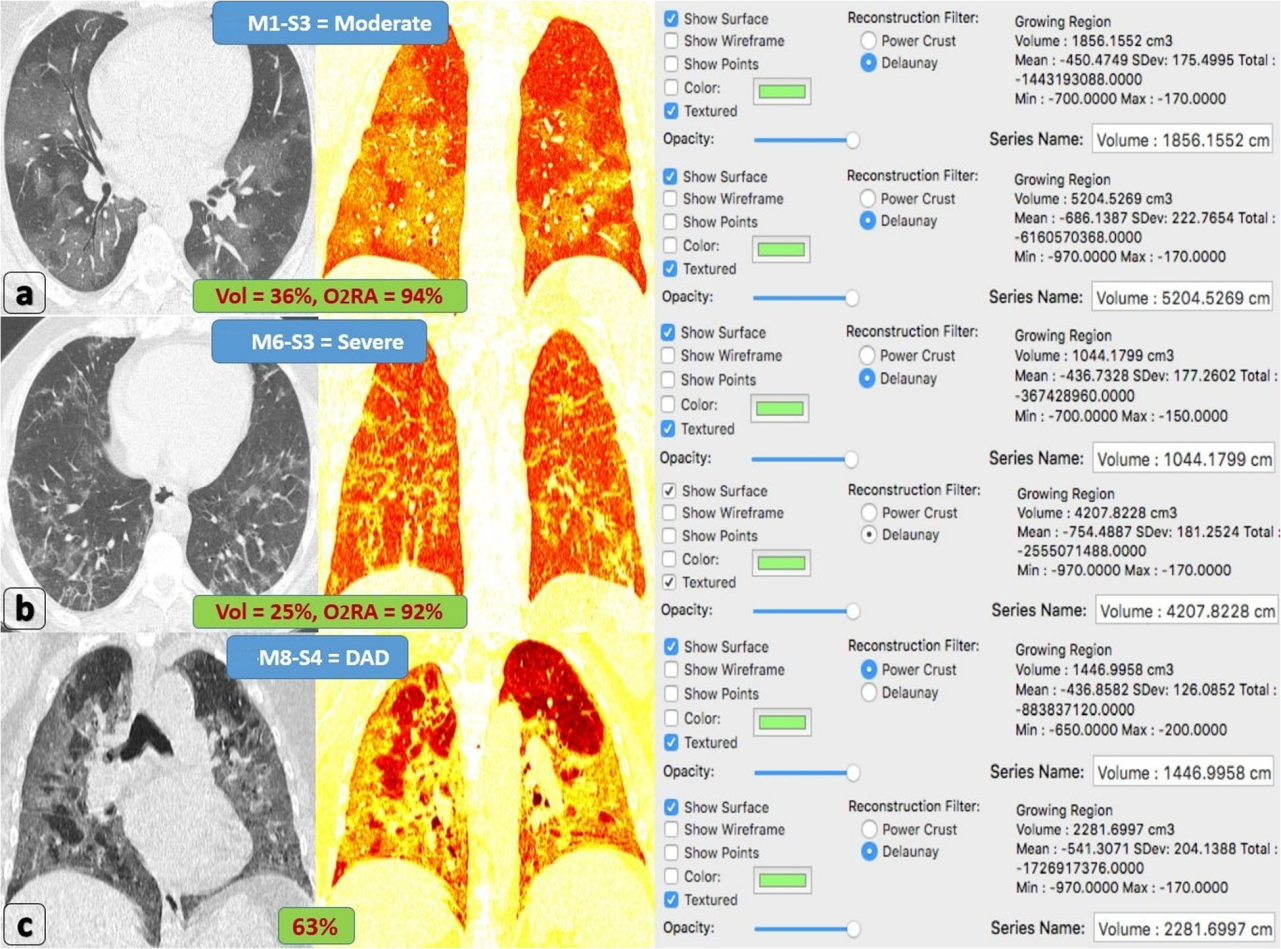
Combined morphologic/volumetric analysis. **a** A 33-year-old male patient proved with COVID-19 complaining of dyspnea, tachypnea, and 94% O_2_RA (consistent with border-line severity). Axial CT lung window and coronal 2D computed volumetric analysis showing bilateral widespread patchy pure GGOs. 3D computed volumetric analysis after threshold interval adjustment revealed 1856 cc pathological lung volume, 5204 cc total lung volume, and 36% lung involvement. Overall CT coding (M1-S3). **b** A 61-year-old male patient proved with COVID-19 complaining of dyspnea, tachypnea, and 92% O_2-_RA (clinically severe). Axial CT lung window and coronal 2D computed volumetric analysis show bilateral patchy GGOs mixed with early fibrotic changes and mild parenchymal distortion. 3D computed volumetric analysis after threshold interval adjustment revealed 1044 cc pathological lung volume, 4207 cc total lung volume and 25% lung involvement. Overall CT coding (M6-S3). **c** A 67-year-old male patient proved with COVID-19 complaining of severe dyspnea and 82% O_2_RA (critical patient indicated for intubation). Coronal CT lung window and coronal 2D computed volumetric analysis showing bilateral diffuse lung involvement with GGOs and super-added septal thickening “crazy-paving pattern.” 3D computed volumetric analysis after threshold interval adjustment revealed 1446 cc pathological lung volume, 2281 cc total lung volume, and 64% lung involvement. Overall CT coding: M8-S4 = DAD

**Fig. 4:**
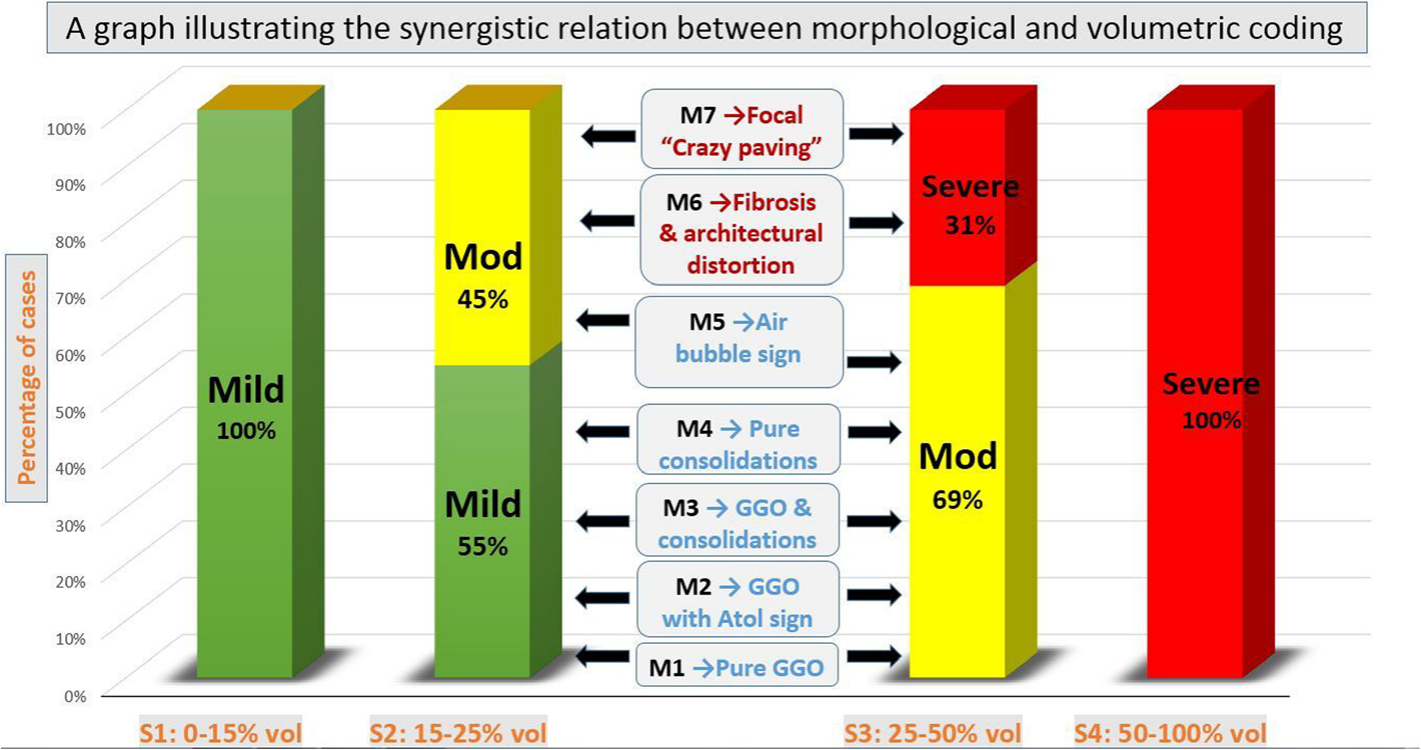
A graph illustrating the additive relation between morphologic and volumetric coding: 100% of [S1] patients are mild whatever their morphological pattern. 100% of [S4] patients are severe whatever their morphological pattern. While 55% of [S2] patients are mild, the remaining 45% are moderate because of the additive effect of [M5, M6 or M7] patterns. While 67% of [S3] patients are moderate, the remaining 33% are severe because of the additive effect of [M6 and M7] patterns

All patients with > 50% lung involvement [S4] were clinically severe or critical. Around 67% of patients with 25–50% lung involvement [S3] were borderline, while the remaining 33% were clinically severe or critical because of the additive effect of either [M6] pattern (early patchy fibrosis with architectural distortion) or [M7] pattern (patchy GGOs with crazy-paving pattern) (Figs. 3 and 4).

**Fig. 5:**
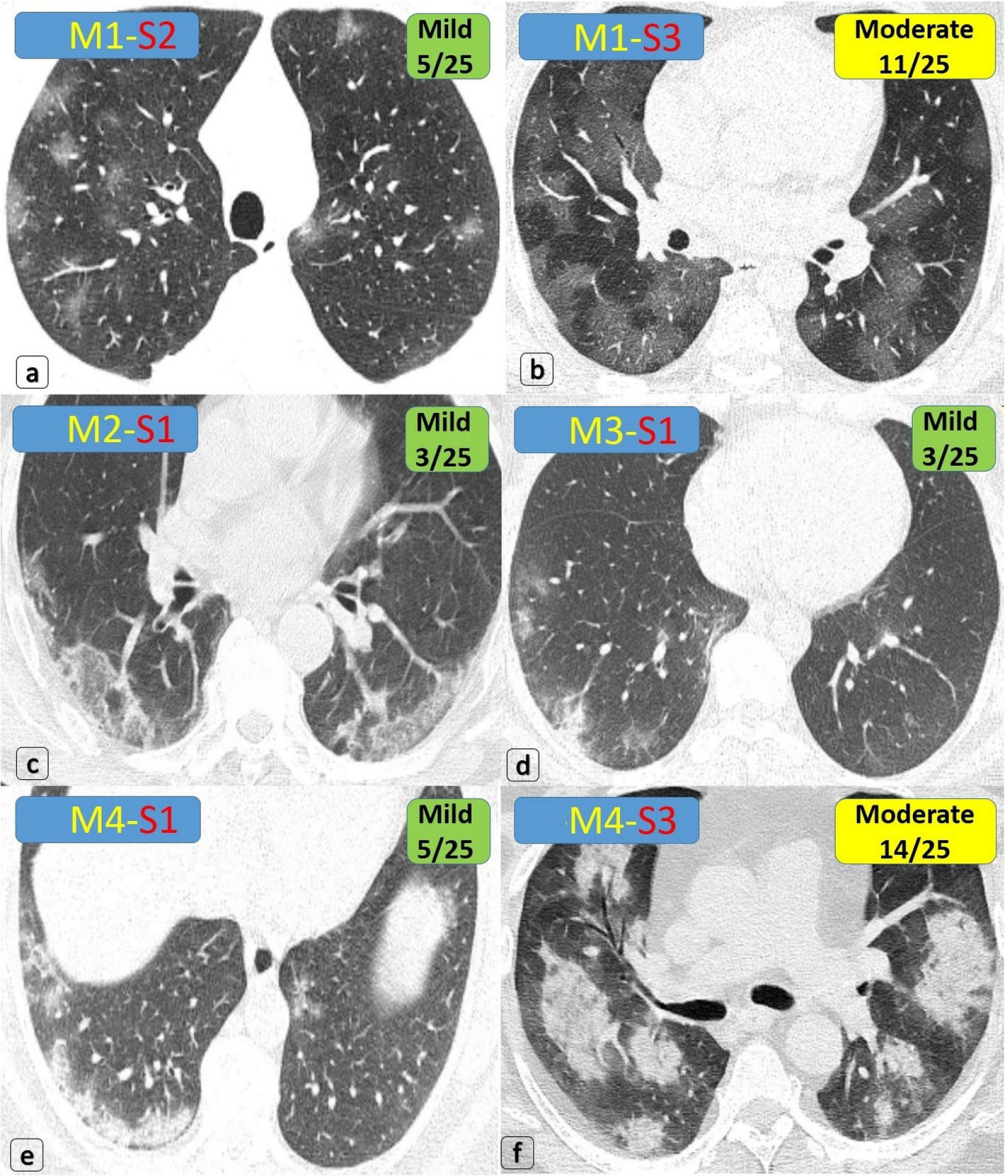
Multiple patients proved with COVID-19, having variable bilateral mild or moderate/border-line lung involvement. **a** Multiple (> 3 in number) small (< 3 cm) bilateral sub-pleural and to lesser extent proximal pure GG nodules (around 5% of lung involvement) … *COV [M1-S2] … CTSI [5/25] … Mild degree*. **b** Bilateral lower lobar large pure ground-glass patches (around 36% of lung involvement) … *COV [M1-S3] … CTSI [11/25] … Moderate degree*. **c** Bilateral lower lobar sub-pleural pure ground-glass patches (> 3 cm in size but only 2 cm deep from the pleural lining) with peripheral “Atoll sign” (around 4% of lung involvement) … *COV [M2-S1] … CTSI [3/25] … Mild degree*. **d** Bilateral scattered small (< 3 cm) GGOs are seen; one of them is mixed with consolidative changes in the right lower lobe (around 3% of lung involvement) … *COV [M3-S1] … CTSI [3/25] … Mild degree*. **e** Right lower lobar curvilinear pattern of consolidations (> 3 cm in size but only 1 cm deep from the pleural lining), a small left lower lobar GGO is seen (around 3% of lung involvement) … *COV [M4-S1] … CTSI [5/25] … Mild degree*. **f** Bilateral multiple variably sized proximal and peripheral (> 3 cm) homogeneous consolidative patches (around 27% of lung volume) … *COV [M4-S3] … CTSI [14/25] … Moderate degree*

All patients with < 15% lung involvement [S1] were mild. Around 53% of patients with 15–25% lung involvement [S2] were mild, while the remaining 47% were borderline because of the additive effect of the [M5] pattern (GGOs with air bubble sign) (Fig. 5)

The overall time for this combined quantitative and morphologic CT assessment was estimated and ranged from 6 to 12 min.

#### A proposed new descriptive CT coding

Based on the combination of morphologic and volumetric analysis, a new descriptive CT code was proposed *[M (1-8)/S (1-4)]*. Distribution prevalence for our patients regarding new CT coding was grouped (Table 3).

**Table 3:** Distribution of patients according to combined morphologic and volumetric CT coding

Mild			Borderline-severe			Severe or critical		
Pattern	***N***	%	Pattern	***N***	%	Pattern	***N***	%
**M1-S1**	115	39%	**M1-S3**	4	7%	**M5-S4**	1	4%
**M1-S2**	35	12%	**M2-S3**	4	7%	**M6-S3**	8	33%
**M2-S1**	40	13%	**M3-S3**	10	18%	**M7-S3**	8	33%
**M2-S2**	6	2%	**M4-S3**	14	25%	**M8-S4**	7	29%
**M3-S1**	64	21%	**M5-S2**	2	4%	**Total**	***24***	***100%***
**M3-S2**	6	2%	**M5-S3**	1	2%			
**M4-S1**	13	4%	**M6-S2**	11	19%			
**M4-S2**	2	1%	**M7-S2**	11	19%			
**M5-S1**	3	1%	**Total**	***57***	***100%***			
**M6-S1**	7	2%						
**M7-S1**	7	2%						
**Total**	***298***	***100%***						

#### A proposed new optimized CTSI (Table 4)

**Fig. 6:**
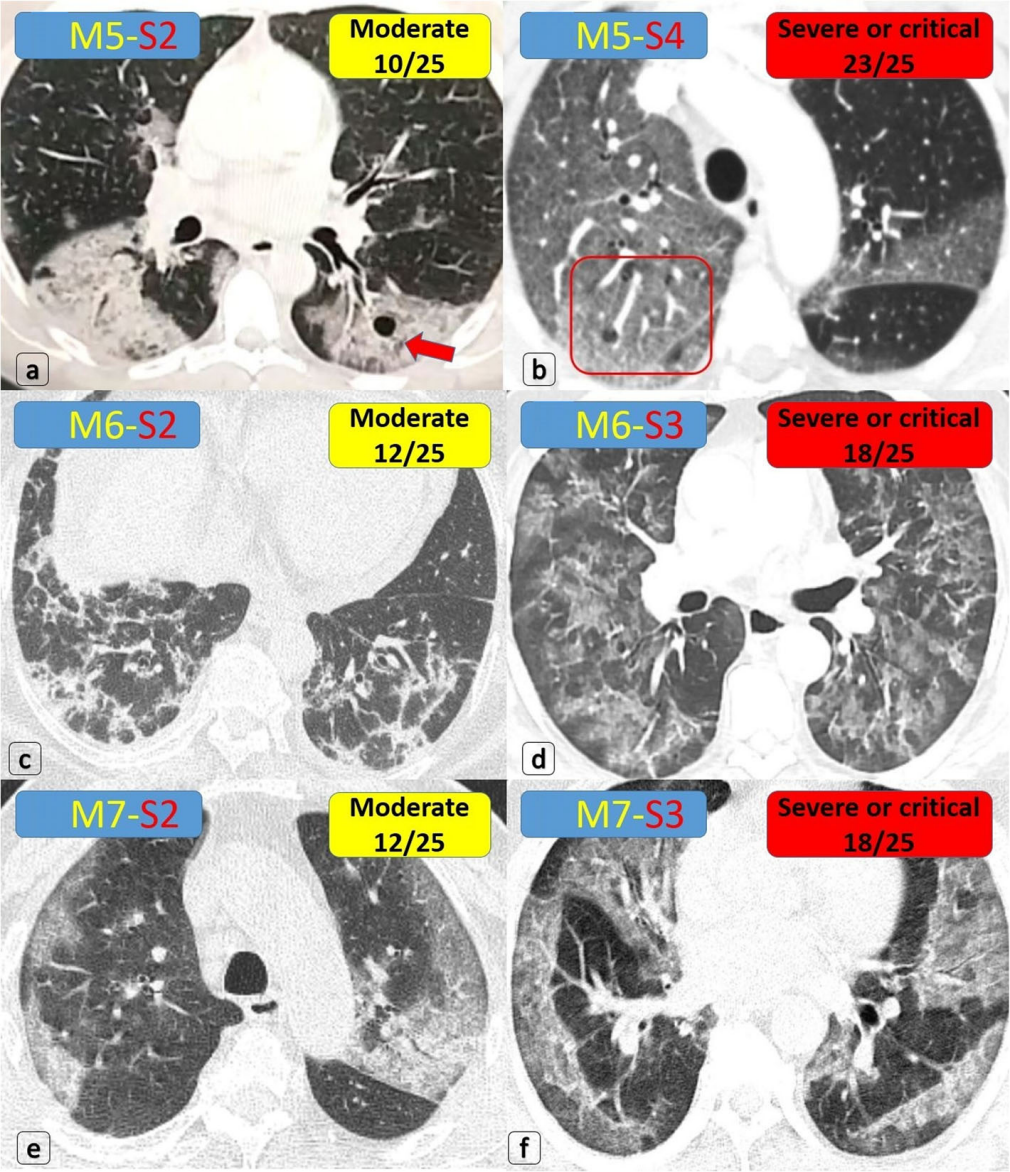
Multiple patients proved with COVID-19, having variable bilateral moderate/border-line or severe/critical lung involvement. **a** Bilateral large GGOs (extending > 3 cm deep from the pleural lining and implicating around 16% of lung volume) show internal air densities (red arrow) representing “air bubble sign” … *COV [M5-S2] … CTSI [10/25] … Moderate degree*. **b** Bilateral large GGOs (involving > 50% of lung volume) with few “air bubble densities”… *COV [M5-S4] … CTSI [23/25] … Severe or critical degree*. **c** Bilateral large early secondary fibrotic changes on top of GGOs with parenchymal distortion (around 19% of lung volume) … *COV [M6-S2] … CTSI [12/25] … Moderate degree*. **d** Bilateral large early secondary fibrotic changes on top of GGOs with parenchymal distortion (around 40% of lung volume) … *COV [M6-S3] … CTSI [18/25] … Severe or critical degree*. **e** Bilateral mid-zonal mainly peripheral patchy GGOs with “crazy paving pattern” (involving around < 18% of lung volume) … *COV [M7-S2] … CTSI [12/25] … Moderate degree*. **f** Bilateral multiple variable-sized patchy proximal and peripheral GGOs with “crazy-paving pattern” (around 40% of lung volume) … *COV [M7-S3] … CTSI [18/25] … Severe or critical degree*

Based on the prevalence and severity analysis, an optimized CTSI is proposed with a “2–25 points” scoring system (Table 4); (1–8 points) are given for the morphologic assessment [M], while (1–17 points) are given for the volumetric/size score [S]. Three categories were achieved corresponding to the clinical severity: (1) mild CTSI (2–9 points), (2) borderline or moderate CTSI (10–17 points), and (3) severe or critical CTSI (18–25 points). A survey for patients regarding new CT coding and new CTSI was demonstrated (Figs. 5 and 6).

**Table 4:**
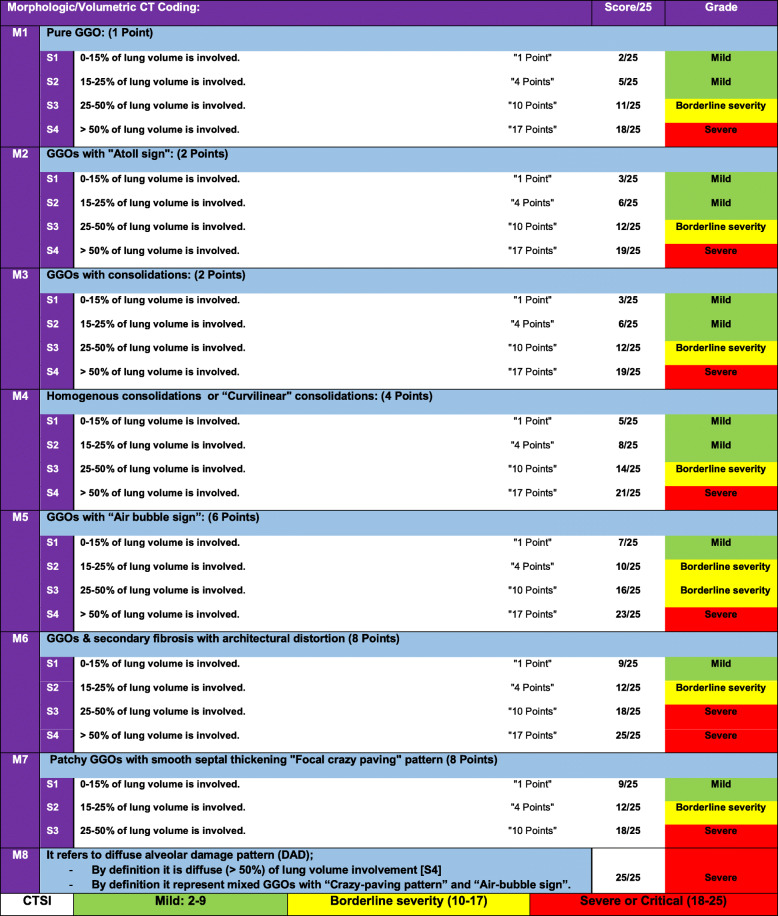
Combined morphologic/volumetric CT coding—new proposed CTSI for COVID-19

#### Evaluation of patient clinical-radiological outcome (14 to 30 days after the onset of symptoms)

All patients with mild clinical presentation showed clinical improvement over the next 14–21 days. Only 104/298 patients (35%) with delayed recovery had followed up CT scans. Radiological improvement was noted among all of them regarding the morphological CT features (depicted by resolution or regression in the density of ground glass or consolidation lesions). Around 91/104 patients (87.5%) also showed regression in quantitative measurements, while 13/104 patients (12.5%) showed the stationary course of which, but with morphological improvement.

37/57 patients (65%) with borderline severity had a follow-up assessment with CT pulmonary angiography protocol after the deterioration of clinical condition into severe/critical status (O_2_ saturation dropped below 93%). The radiological progressive course was depicted regarding both quantitative measurements and morphological features of parenchymal fibrosis and crazy paving pattern. One patient who had a previous “air bubble sign” developed pneumothorax. The vascular assessment was unremarkable. Thirty-one patients received high flow nasal oxygen while 6 patients needed mechanical ventilation later on.

22/24 critical patients (91.7%) showed deterioration in the clinical condition regarding the O_2_ requirements; 20 patients needed mechanical ventilation while two patients had sudden death before ventilation. Only bed-side X-ray was used for follow-up of these patients. Two patients only had partial improvement regarding O_2_ saturation and requirements and follow-up CT scans later on revealed regression regarding both quantitative and morphological CT features.

### Step (2): standardized blinded/independent validation analysis for the proposed new CTSI

**Fig. 7:**
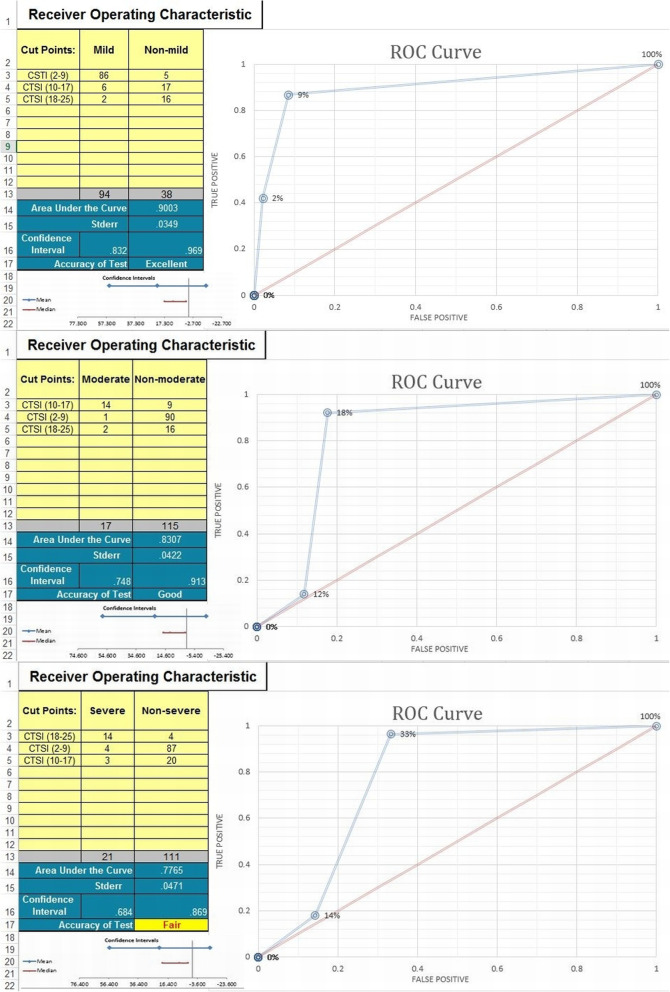
Receiver operating characteristics (ROC) curves describing the accuracy results for the validation analysis of the proposed CTSI (ranging from fair to excellent)

Accepted inter-observer agreement (IOA) was achieved (ranging from 81.3%:100%). High accuracy for the proposed new CTSI was reached (ranging from 90.1%:91.7%). High degrees of specificity and NPV were estimated (ranging from 86.8%:96.4% and 80.5%:97.2%, respectively). Moderate to a high degree of sensitivity and PPV were estimated (ranging from 66.7%:91.5% and 60.9%:94.5%, respectively). Based on AUROC curve measurements (ranging from 0.7765: 0.9003) and confidence intervals (ranging from 0.684:0.832), the proposed CTSI expressed excellent, good, and fair validity for evaluation of mild, borderline, and severe patients, respectively. A detailed review of all validation analysis measurements was grouped (Table 5) and demonstrated (Fig. 7).

**Table 5:** Summary of the statistical results regarding “step 2 blinded validation analysis”

	Mild CTSI	Moderate CTSI	Severe CTSI	
**Mild clinical disease**	**86** (91%)	6	2 (with patchy crazy-paving and early fibrosis)	Total (disease) = **94**
**Borderline severe clinical disease**	1	**14** (82%)	2 (with patchy crazy-paving and early fibrosis)	Total (disease) = **17**
**Severe or critical disease**	4	3	**14** (67%)	Total (disease) = **21**
**Total (CT)**	**91** (69%)	**23** (17%)	**18** (14%)	Total = **132**
**Prevalence**	*71.2%*	*12.9%*	*15.9%*	
**Sensitivity**	*91.5%*	*82.4%*	*66.7%*	*Mean = 80.2%*
**Specificity**	*86.8%*	*92.2%*	*96.4%*	*Mean = 91.8%*
**PPV (positive predictive value)**	*94.5%*	*60.9%*	*77.8%*	*Mean = 77.7%*
**NPV (negative predictive value)**	*80.5%*	*97.2%*	*93.9%*	*Mean = 90.5%*
**IOA (inter-observer agreement)**	*87.5%*	*81.3%*	*100%*	*Mean = 89.6%*
**AUC (area under curve)**	*0.9003*	*0.83007*	*0.7765*	*Mean = 0.8356*
**Confidence interval**	*0.832*	*0.748*	*0.684*	*Mean = 0.7546*
**Accuracy**	*90.1%*	*90.9%*	*91.7%*	*Mean = 90.9%*
**Validity of the test**	*Excellent*	*Good*	*Fair*

## Discussion

Clinical confusion was aroused about COVID-19 patients who had similar quantitative CT measurements and variable clinical prognosis. The presence of pre-existing comorbidities or vascular angiopathy is known clinical and radiological explanations but they could be absent. The authors suggested another radiological explanation by adding morphological severity to the volumetric severity.

This study was different from previous studies which only stressed the quantitative lung assessment and did not exclude those confounding parameters; such as Yang et al. [10], Lessmann et al. [11], and Li et al. [12]. Leonardi et al. [13] excluded only pleuro-parenchymal lung diseases. On the other hand, authors agreed with studies that pointed to the impact of some morphological patterns on clinical severity, such as Li et al. [14] and Zhao et al. [15], but they did not correlate them to CT severity scoring. Qin et al. [16] put isolated quantitative severity score beside dedicated severity score for “crazy paving pattern” and consolidations.

In this study, all patients with > 50% lung involvement were clinically severe/critical. This is precisely keeping with Li et al. [12] and generally matching with previous studies which focused on the relation between the pathological lung volume and clinical severity.

Leonardi et al. [13] reported that the critical value for quantitative lung assessment was 23%. In this study, it was nearly similar (25%). Still in this study, the authors found that only 33% of [S3] patients were severe/critical because of an additive effect of early secondary fibrosis patchy crazy-paving.

In this study, the “crazy-paving” pattern was highly associated with clinical severity, this is matching Qin et al. [16], Lyu et al. [17], and Hu et al. [18] who correlated it to diffuse alveolar damage (DAD). Moreover, the authors found an additive relation of which to the clinical severity among [S2] and [S3] patients.

This study also agreed with Li et al. [14], Zhao W et al. [15], Qin et al. [16]^,^ and Hu et al. [18] that early fibrosis and architectural distortion accompanied severe patients.

Authors believed that early fibrosis which may occur during the first 10 days of the disease is different from delayed fibrosis during the natural healing process. Early fibrosis presents by irregular fibro-atelectatic bands; perpendicular to the pleural surface with diminished volume. While the delayed fibrosis of healing presents by curvilinear bands parallel to the pleural surface with sub-pleural sparing. In this study, early fibro-atelectatic bands with architectural distortion additively increased clinical severity in [S3] patients. This could solve any dilemma between Pan et al. [7] who suggested a good prognosis of fibrosis and Pan et al. [19] as well as Kong et al. [20] who suggested a bad prognosis of which.

Nearly similar to Yee et al. [21], the “air bubble sign” was found among 2% of our patients. Furthermore, in this study, it was associated with increased severity among [S2] patients. This is mostly explained by associated fibrotic changes.

Also, similar to Zhao et al. [15] and Hu et al. [18], the severity rate increases in proportionate to the degree of consolidative changes.

The current proposed COVID-19 CTSI expressed accepted results after blinded validation analysis.

This study has the merit that it was conducted on a large group of patients. Meanwhile, the above-mentioned studies were all conducted on smaller groups (ranging from 78:150 patients). The overall time for every HRCT evaluation along with CTSI estimation and CT reporting was also very reasonable and did not exceed 12 min.

However, this study was limited by the low percentage of severe cases in addition to the limited clinical indication/value for CT assessment of mechanically ventilated patients.

## Conclusion

Based on severity analysis, HRCT morphological features should share in CT severity scoring of COVID-19 patients (notably the pulmonary fibrosis and “crazy-paving pattern”). A new optimized CTSI with accepted validation is proposed for the initial staging of COVID-19 patients, using combined morphologic/volumetric assessment instead of the quantitative assessment alone. It could solve the clinico-radiological mismatch among patients with similar quantitative CT results and variable clinical presentation during the absence of pre-existing comorbidities or vascular complications.

## Data Availability

The datasets used and/or analyzed during the current study are available from the corresponding author on reasonable request.

## Abbreviations

COVID 19: Corona virus disease 2019
CTSI: Computed tomography severity index
GGOs: Ground glass opacities
HRCT: High-resolution computed tomography
RT-PCR: Reverse transcription polymerase chain reaction
O_2_ Sat/Ra: Oxygen saturation in room air
DAD: Diffuse alveolar damage
